# Characterization and Modeling Analysis for Metal-Semiconductor-Metal GaAs Diodes with Pd/SiO_2_ Mixture Electrode

**DOI:** 10.1371/journal.pone.0050681

**Published:** 2012-11-30

**Authors:** Shih-Wei Tan, Shih-Wen Lai

**Affiliations:** Department of Electrical Engineering, National Taiwan Ocean University, Keelung, Taiwan, Republic of China; Florida International University, United States of America

## Abstract

Characterization and modeling of metal-semiconductor-metal (MSM) GaAs diodes using to evaporate SiO_2_ and Pd simultaneously as a mixture electrode (called M-MSM diodes) compared with similar to evaporate Pd as the electrode (called Pd-MSM diodes) were reported. The barrier height (*φ*_b_) and the Richardson constant (A*) were carried out for the thermionic-emission process to describe well the current transport for Pd-MSM diodes in the consideration of the carrier over the metal-semiconductor barrier. In addition, in the consideration of the carrier over both the metal-semiconductor barrier and the insulator-semiconductor barrier simultaneously, thus the thermionic-emission process can be used to describe well the current transport for M-MSM diodes. Furthermore, in the higher applied voltage, the carrier recombination will be taken into discussion. Besides, a composite-current (CC) model is developed to evidence the concepts. Our calculated results are in good agreement with the experimental ones.

## Introduction

The metal-semiconductor (MS) contact and the metal-oxide-semiconductor (MOS) capacitor are the most useful device in the study of semiconductor surfaces and essential component in semiconductor device. MS contact with rectifying characteristic is widely used in MESFETs, HEMTs, optical sensors, and gas sensors. MOS capacitor with voltage-controlled variable is used in MOSFETs for forefront high-density integrated circuits [1]–[4]. Recently, Hydrogen has been widely used in hydrogen-fueled vehicles, medical treatment, chemical industry, and semiconductor fabrication. However, hydrogen-containing gases have the risk to cause explosion. Therefore, the development of hydrogen sensors for real-time in situ detection is highly required. A number of palladium and platinum-based hydrogen sensors have been demonstrated [5]–[22]. Among them, MS diodes [5]–[13] have been addressed to be one of the most promising devices. Hydrogen sensors employing MOS diodes have also been extensively studied [14]–[18].

In addition, Chiu et al. [19]–[22] reported a new MSM hydrogen sensor with two multifinger Schottky contacts. Unlike conventional MS and MOS diodes, a mixture of palladium and silicon dioxide (SiO_2_) is deposited upon the semiconductor layer. Compared to commonly used MS and MOS diodes, M-MSM diodes obtained excellent performance of high sensitivity. However, the current–voltage (I–V) curve represents the diode current operated as sensor in N_2_. I-V curve for M-MSM diodes differ from one for MS diodes in that the former exhibit the multiple-step phenomenon, while the latter are not. The reason of causing the multiple-step phenomenon is very interesting but there are no descriptions in Chiu et al. reported [22]. In this paper, characterization and modeling of M-MSM GaAs diodes were reported. The *φ*_b_ and the A* were determined by a deduced equations from the I-V curve that operated at various temperature. The carrier over both the metal-semiconductor barrier and the insulator-semiconductor barrier are considered simultaneously on the thermionic emission process that can be used to describe well the current transport for M-MSM diodes. With increasing the applied voltage, the number of minority carrier at the semiconductor surface is larger than of the majority carrier. The carrier recombination will be taken into consideration. Furthermore, a composite current (CC) model is developed to evidence the concepts. The calculated results are in good agreement with the experimental ones. Finally, conclusions were made.

### Device Structure and Fabrication

**Figure 1:**
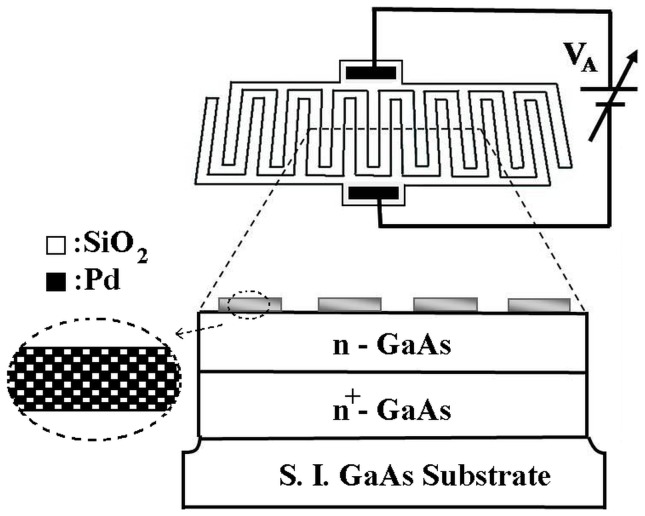
Schematic view of a M-MSM diodes with two multi-finger Pd/SiO_2_ mixture electrodes.

The epitaxial structure was grown on a (100)-oriented GaAs substrate by LP-MOCVD. It consisted of a 0.6 µm n^+^-GaAs layer, and a 0.8 µm n-GaAs layer with 8×10^16^ cm^−3^ doping concentration. The process started with mesa isolation. HCl was used to remove the native oxide on the 0.8 µm n-GaAs layer after a device mesa. Two multiple-fingers Schottky electrodes forming a MSM diodes were implemented by thermally depositing a 30 nm mixture with various weight-ratios of Pd to SiO_2_. Both the finger width and the finger-to-finger spacing are 5 µm. The area of the multiple-fingers electrode was A ≈ 8×10^−4^ cm^2^. Another MSM diodes with a 30 nm Pd directly deposited upon the GaAs layer was also fabricated for comparison. Device measurement was carried out by a custom-made 235 ml flow-through test chamber made from stainless steel and filled with the 99.99% nitrogen gas at a flow rate of 500 sccm. Fig. 1 shows the schematic views for the finally fabricated M-MSM diodes.

**Figure 2:**
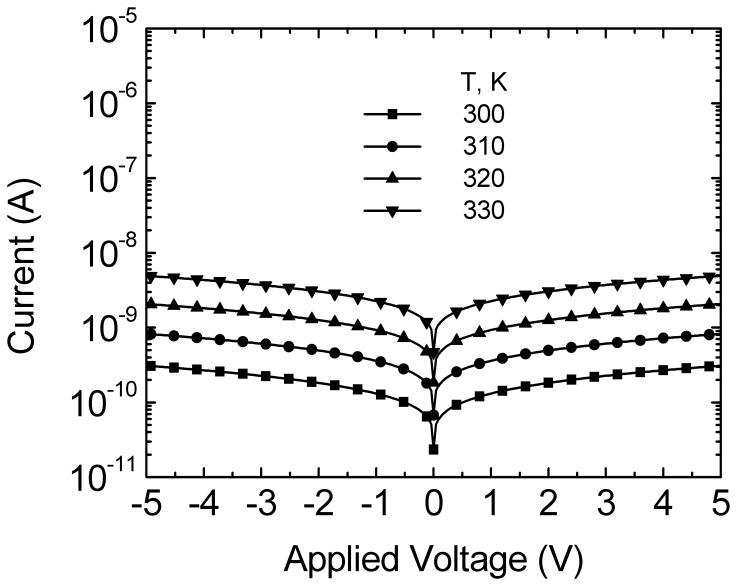
Currents as a function of applied voltage for Pd-MSM diodes at various temperatures. Calculated data are included.

### Determination of Barrier Height and Richardson Constant

I–V curves of Pd-MSM diodes at various temperatures in the range of 300 K to 330 K are shown in Fig. 2. The solid symbols are the calculated results. Because the quality of the epitaxial wafer and the evaporative Pd are excellent and uniform, all curves indicate bidirectional and symmetrical. The thermionic-emission process for carrier and the image-force lowering are considered simultaneously on the current of Pd-MSM diodes (I_Pd_), I_Pd_ can be expressed as [23].
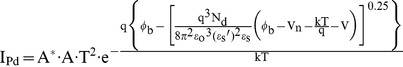
(1)where A* = 8.9 A/k-cm^2^ is the Richardson constant for GaAs and be given by
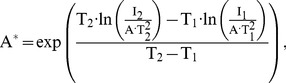
(2)and *φ*_b_ = 0.80 eV is the barrier height and be given by
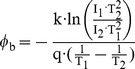
(3)Other parameters of A ≈ 8×10−4 cm2, T = 300 K to 330 K, q = 1.6×10−19 C [23], k = 1.38×10−23 J/K [23], N_d_ = 8×1016 cm−3, ε_o_ = 8.85×10−14 F/cm [23], ε_S_’ = 10.8, ε_S_ = 12.9 [23], Vn = 0.05 V, V = 0 V to 5V are the contact area, an absolute temperature, the unit electronic charge, the Boltzmann constant, the doping concentration, the permittivity of free space, the relative permittivity of GaAs near the Pd, the relative permittivity of GaAs, the Fermi potential from conduction-band edge, and an applied voltage, respectively. Following the previous article [24], the electron approaches the metal with the thermal velocity, and one might except that there is not enough time for the semiconductor to become fully polarized by the electric field, so that ε_S_’ is less then ε_S_. For our calculation in Fig. 2, the results are also represented with a good agreement found. This means that the I_Pd_ together with the extracted device-parameters is very promising for well describing for Pd MSM diodes behaviors.

**Figure 3:**
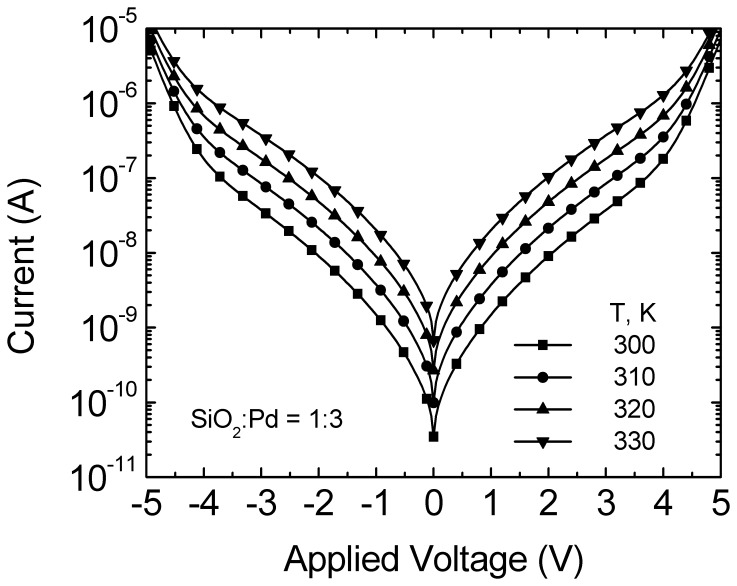
Currents as a function of applied voltage for M-MSM diodes with weight-ratio of SiO_2_ and Pd equal to 1∶3 in mixture at various temperatures. Calculated data are included.

**Figure 4:**
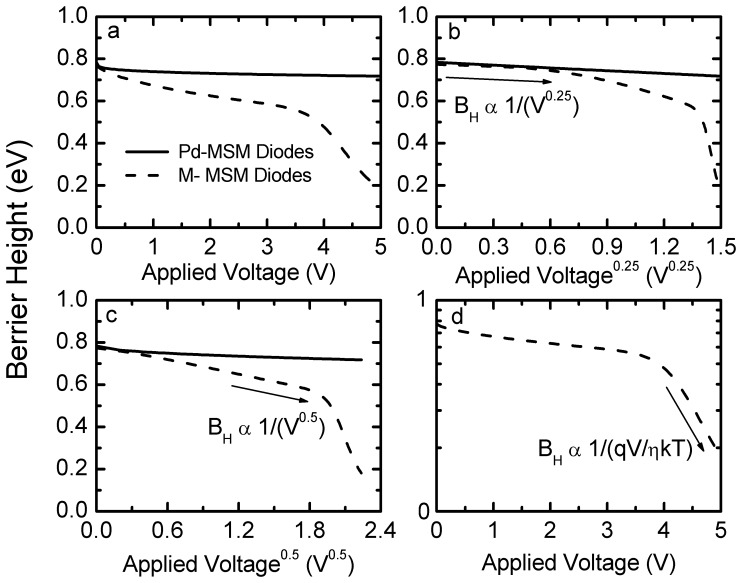
Barrier height (*φ*_b_) as a function of (a) applied voltage, (b) applied voltage to the power of 0.25, (c) applied voltage to the power of 0.5, and (d) ln*φ*_b_ as a function of applied voltage for a Pd-MSM diodes (solid lines) and a M-MSM diodes (dashed lines).

**Figure 5:**
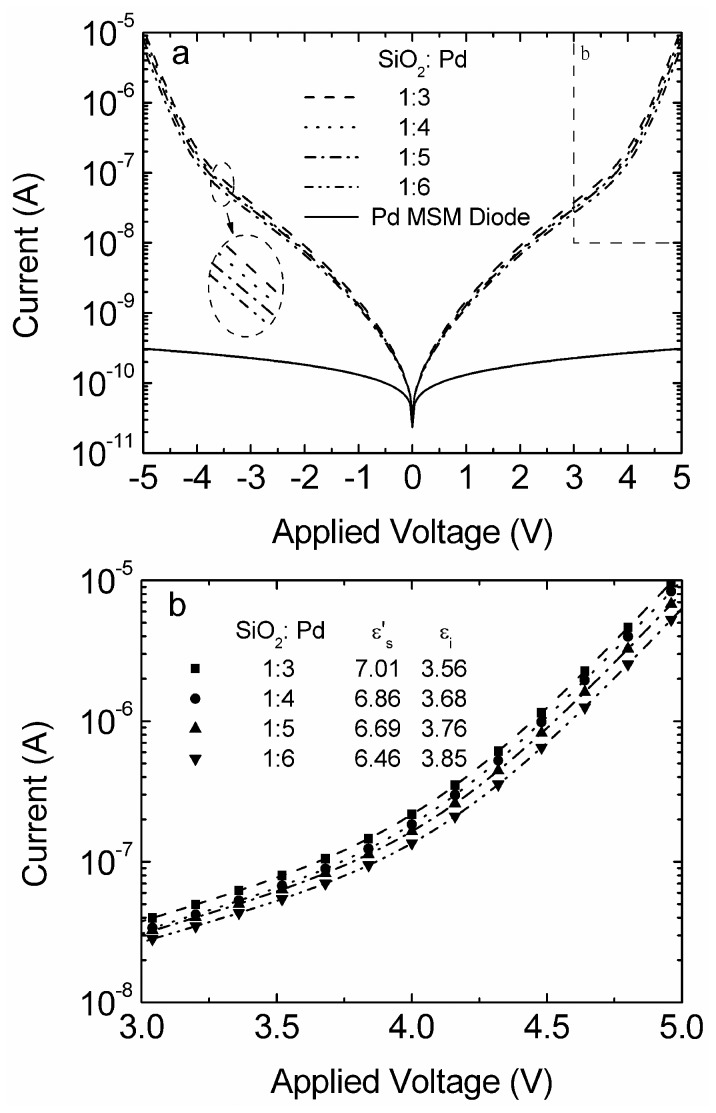
Currents as a function of applied voltage for M-MSM diodes with various weight-ratios of SiO_2_ and Pd at 300 K. Calculated data are included.

### Experimental Performance and Modeling Deducing

Unlike the Fig. 2 represented I_Pd_, Fig. 3 shows I–V curves with a multiple-step phenomenon of M-MSM diodes with the mixture electrodes in the weight-ratio of SiO_2_ to Pd equal to 1/3 at various temperatures in the range of 300 K to 330 K. The solid symbols are the calculated results. In order to probe into the multiple-step phenomenon in I–V curves, Fig. 4(a) shows the *φ*_b_ as a function of the applied voltage for M-MSM diodes with the mixture electrodes in the weight-ratio of SiO_2_ to Pd equal to 1/3, *φ*_b_ as function of applied voltage for Pd-MSM diodes is also included for comparison. The line slope,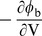, shows the multiple-step phenomenon and indicates that the carrier over both the metal-semiconductor barrier and the insulator-semiconductor barrier together with image-force lowering are considered on the thermionic emission process. That is, to notice Fig. 4 (b), *φ*_b_ against V^0.25^ represents a straight line from V^0.25^ = 0 V to 0.58 V. Hence, the current component of the thermionic-emission process for carrier over the metal-semiconductor barrier (I_MS_) is considered on the CC model for M-MSM diodes (I_M_), I_MS_ can be expressed as
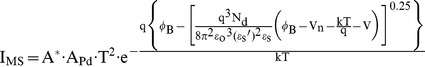
(4)where A* = 9.6 A/k-cm^2^ and *φ*_B_ = 0.81 eV are given by Eq.2 and Eq.3, respectively. A_Pd_≈2.90×10^−4^ cm^2^ and ε_S_’ = 7.01 are the effective Pd-contact area and the relative permittivity of GaAs near the mixture, respectively. Other parameters are the same as previous. Similarly, to notice Fig. 4 (c), *φ*_b_ against V^0.5^ represents a straight line from V^0.5^ = 1.2 V to 1.9 V. For that reason, the current component of thermionic-emission process for carrier over the insulator-semiconductor barrier (I_MIS_) is considered on I_M_, I_MIS_ can be expressed as [23]

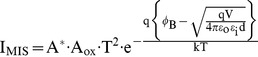
(5)where the effective oxide-contact area is A_ox_≈5.10×10−4 cm2. The ε_i_ = 3.56 [23] and the d = 30 nm are the relative permittivity and the thickness of mixture, respectively. Other parameters are the same as IMS. To notice Fig. 4 (d), ln*φ*_b_ against V represents a straight line with the applied voltage larger then 4 V. When a larger voltage is applied (>4 V), the bands bend even more downward so that the intrinsic level Ei at the surface crosses over the Fermi level EF. At this point the number of holes (minority carriers) at the surface is larger then that of the electrons, the thermionic-emission of electrons will be recombined by holes and the current is proportional to qV/ηkT. Therefore, the current component of recombination (IRB) is considered on IM. The current IRB can be expressed as [23]

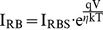
(6)where IRBS = 4.81×10−16 A, and η = 8.1 are the saturation current of recombination, and an ideality factor, respectively. Then, IM can be approximated by the sum of Eqs. 4, Eqs. 5, and Eqs. 6. In Fig. 3, calculated results at various temperatures are also included with a good agreement found. This means that IM together with the extracted parameters is very promising for well describing M-MSM diodes behaviors.

**Figure 6:**
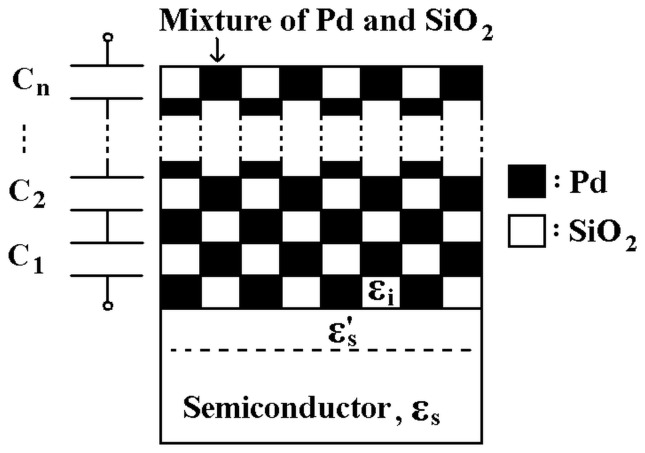
Schematic view of Pd/SiO_2_ mixture electrode for M-MSM diodes to calculate the effective relative permittivity.

**Figure 7:**
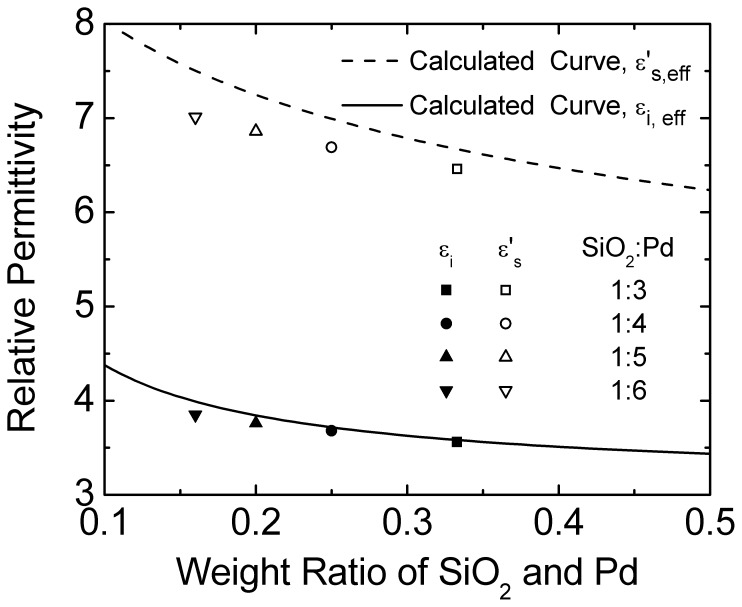
Calculated relative permittivity as a function of weight-ratio of SiO_2_ and Pd. Fitting data for experimental I-V curve are included.

Figure 5(a) shows I–V characteristics of M-MSM diodes with the mixture electrodes in various weight-ratios of SiO_2_ to Pd. I–V characteristic of Pd-MSM diodes is also shown for comparison. I_M_ that were marked by solid symbol together with the extracted parameters are shown in Fig. 5(b). I_M_ together with the extracted parameters is very promising for well describing the experimental results.

On the other hand, ε_s_’ and ε_i_ associated with the mixture in various weight-ratios of SiO_2_ to Pd are the key parameters and play an important role on the performance of M-MSM diodes. For simplifying the calculation of the relative permittivity, the composition of mixture is uniform for assumption. Fig. 6 shows the schematic view of Pd/SiO_2_ mixture electrode for M-MSM diodes. ε_s_’ is proportional to the ratio of A_Pd_ and A_ox_. So the effective relative permittivity of GaAs near the mixture (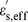) can be calculated as
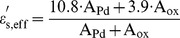
(7)


Consideration of equivalent circuit, the capacitance of mixture can be expressed as
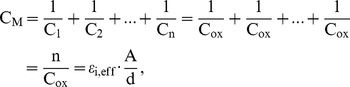
where 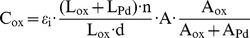 is the capacitance of SiO_2_, L_pd_, L_ox_, A, and d are the Pd thickness, the SiO_2_ thickness for mixture, the contact area, the thickness of mixture, respectively. Then the effective relative permittivity of mixture (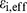) can be deduced to



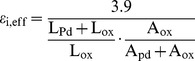
(8)

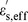
 and 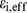 are shown in Fig. 7. ε_s_’ and ε_i_ are also shown for comparison. Consideration of boiling point of Pd (2963°C) more then SiO_2_ (2230°C), the actual weight-ratio of SiO_2_ and Pd is larger than the prepared weight-ratio of SiO_2_ to Pd after evaporation.

### Conclusions

In summary, characterization and modeling of MSM GaAs diodes using to evaporate SiO_2_ and Pd simultaneously as the mixture electrode were investigated. Effects of operating at various temperatures and a mixture with the various weight-ratios of SiO_2_ to Pd on electrical performances were investigated. *φ*_b_ and A* were determined to the thermionic emission process to describe well the current transport for Pd-MSM diodes in the consideration of the carrier over the metal-semiconductor barrier. In addition, in the consideration of the carrier over both the metal-semiconductor barrier and the insulator-semiconductor barrier simultaneously, thermionic emission process can be used to describe well the current transport for M-MSM diodes. Furthermore, in the higher applied voltage, the number of minority carriers at the semiconductor surface is larger then of the majority carrier. The carrier recombination will be taken into discussion. Besides, I_M_ was developed to evidence the concepts. Our calculated results are in good agreement with the experimental ones.
